# Longitudinal association of dietary sources of animal and plant protein throughout childhood with menarche

**DOI:** 10.1186/s12887-021-02670-8

**Published:** 2021-04-28

**Authors:** Nazanin Moslehi, Golaleh Asghari, Parvin Mirmiran, Fereidoun Azizi

**Affiliations:** 1 Nutrition and Endocrine Research Center, Research Institute for Endocrine Sciences, Shahid Beheshti University of Medical Sciences, No. 24, Shahid Arabi St, Yemen Blvd, Chamran Exp, P.O.Box: 19395-4763, Tehran, Iran; 2 Department of Clinical Nutrition and Dietetics, Faculty of Nutrition and Food Technology, National Nutrition and Food Technology Research Institute, Shahid Beheshti University of Medical Sciences, No. 7, Arghavan-e-gharbi St., Farahzadi Blvd., Shahrak-e-qods, P.O.Box: 19395-4741, Tehran, Iran; 3 Endocrine Research Center, Research Institute for Endocrine Sciences, Shahid Beheshti University of Medical Sciences, Tehran, Iran

**Keywords:** Plant protein, Animal protein, Menarche, Dairy, Meat

## Abstract

**Background:**

Inconsistent findings have been reported for associations between protein intake and age at menarche. We aimed to investigate the association between intake of protein and protein-containing food groups during childhood with menarche among Iranian girls.

**Methods:**

Girls aged 6–18 years who did not experience menarche in the third or fourth examination cycles of the Tehran Lipid and Glucose Study were selected and were followed to the fifth examination cycle. Daily intakes of protein from different animal/plant sources and ten protein-containing food groups were assessed using a food frequency questionnaire at baseline (the third or fourth examination cycles). Occurrence of menarche and its onset age were asked during each examination cycle. Cox proportional hazards regression was used to estimating hazard ratios (HRs) and 95% confidence interval (95%CI) for the occurrence of menarche per one standard deviation (SD) of dietary intakes. Logistic regression was also used to estimate the odds of reaching menarche ≤12 years.

**Results:**

During the study, 147(61%) of girls reached menarche, the median age at menarche was 12 years. The adjusted HRs (95% CI) for the occurrence of menarche per one-SD of dietary intakes were 0.68 (0.48, 0.98; *p* = 0.040) for plant protein and 1.36 (1.01, 1.84; *p* = 0.042) for animal protein after adjusting for baseline age, BMI Z-score, energy intake, and mother’s early menarche and education. Substituting 10-g animal protein with plant protein was associated with a 16% (95%CI: 5–25%; *p* = 0.006) lower risk of menarche. Dietary intakes of poultry (adjusted HR: 1.35; 95% CIs: 1.00–1.82; *p* = 0.049) and low-fat dairy (adjusted HR: 1.20; 95%CIs: 0.99–1.46; *p* = 0.064) were marginally associated with the increased risk of menarche. However, the odds of early menarche was significant only for plant protein (adjusted odds ratio = 0.39; 95% CI: 0.16–0.96; *p* = 0.040).

**Conclusions:**

Our findings indicate that the risk of menarche increases by higher intakes of animal protein and decreases by plant protein. Intakes of poultry and low-fat dairy associate with a higher risk of menarche. The odds of menarche occurrence ≤12 years reduces by higher intakes of plant protein.

**Supplementary Information:**

The online version contains supplementary material available at 10.1186/s12887-021-02670-8.

## Background

Menarche, as the late stage of reproductive development, represents sexual maturation in girls [1]. In recent years, the average age at menarche (AAM) has decreased worldwide due to an improved lifestyle and nutritional status [2]. In Iran, a reduction of 0.15 years per decade in menarcheal age was estimated between 1930 and 1990 [3]. The age when an adolescent girl first begins menses affects their sexual and reproductive health as well as non-reproductive health [4]. Earlier age at menarche has been associated with a higher risk of metabolic syndrome [5, 6], type 2 diabetes [7, 8], hormone-related cancers [9, 10], cardiovascular disease, and mortality later in life during adulthood [11]. In Iranian women, the risk of pre-diabetes, diabetes, and metabolic syndrome was 2.3–3.6 higher in women with menarcheal age < 11 than those with menarcheal age 13–14 years [6, 8]. It has also been estimated that the risk of type 2 diabetes is reduced by 9% per 1-year later occurrence of menarche [7], and the risk of metabolic syndrome is increased by 8% per 1-year decrease in AAM [5].

Although genetic factors are the main determinants of timing of menarche, non-genetic variables such as dietary intakes may provide clues on early intervention to prevent menarche at early ages [12, 13]. Studies on associations of nutritional factors with AAM have a long history, yet few of the menarche’s dietary determinants have been characterized. Considering dietary sources of protein intake, prospective studies conducted in US and German girls suggested an inverse association for animal protein and a positive association for plant protein with AAM [14, 15]. The positive association between animal protein intake and AAM was also reported in girls living in south-west England, while plant protein intake was not associated with menarche [16]. Of protein-containing food groups, dietary intakes of dairy and meats have been mostly investigated, particularly in the Western countries [16–21]. However, the findings of the studies are highly inconsistent. Apart from null results [16, 20], dairy intake was associated with either a lower AAM [18] or a higher AAM [19, 21]. Red meat consumption in south-west England and Colombian girls were reported to be associated with a lower AAM [16, 20], while in the US girls, red meat intake was not related to the likelihood of attaining menarche [19]. Besides, the other dietary sources of protein intake have been less studied concerning AAM. Differences in the availability of foods, cooking methods, and dietary pattern across geographical regions may influence the nutritional determinants of menarche. Since there have been scarce studies in the Middle East and North Africa region, we examined whether total protein, protein intake from animal/plant sources, and specific protein-containing food groups are associated with menarche among Iranian girls.

## Methods

### Participants

**Fig. 1:**
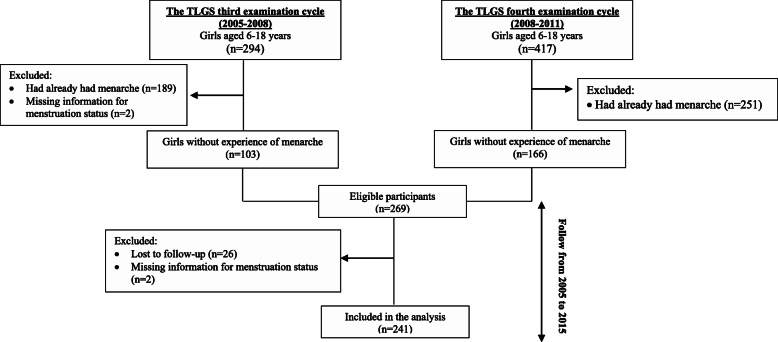
Flow diagram of the participants’ selection

The Tehran Lipid and Glucose Study (TLGS) is a prospective population-based study started in 1999 with 15,005 individuals aged ≥3 years. The participants were selected randomly from residents of district No. 13, Tehran, the capital of Iran. Extensive information on participants’ socio-economic and health has been collected at baseline and updated every 3-years after that [22]. In this study, all girls aged 6–18 years at either the TLGS third (2005–2008; *n* = 294) or the fourth (2008–2011; *n* = 417) examination cycles were selected. After exclusion of those who had already had menarche (*n* = 440) and those with missing information for menstrual status (*n* = 4) or lost to follow-up (*n* = 26), 241 girls were followed to the fifth examination cycle (2012–2015; Fig. 1).

This study was conducted following the principles of the Declaration of Helsinki; written informed consent was obtained from parents or primary caregivers of all girls.

### Data collection

#### Age at menarche

During each examination cycle of the study, the menstruation status of females was assessed using a questionnaire. Girls were asked whether they reached menarche and if yes, the age at which they had attained the menarche was asked in complete years. Maternal menarcheal age was determined based on their self-reporting of the age of initiation of menstruation. The occurrence of menarche at ≤12 years was defined as early menarche based on the median age of menstruation onset of girls. The age of 12 years was also consistent with the AAM of Tehranian girls in TLGS minus one standard deviation (SD) [3].

#### Dietary intakes

Dietary intakes were assessed by a food frequency questionnaire (FFQ) collected either at the third or fourth examination cycles (Supplementary file 1). The frequency and amount of each food item consumed during the past year were assessed using the FFQ and converted to g/day. Intakes of energy and nutrients were determined using the United States Department of Agriculture (USDA) and the Iranian food composition tables [23, 24]. Intakes of total protein, protein from animal and plant sources, and intake of 10 protein-containing food groups including fast food, red meat, poultry, fish, organ meat, egg, legumes, low-fat dairy, high-fat dairy, and total dairy were estimated.

#### Demographic and anthropometric data

Girls’ ages were determined according to their birth date. Maternal education was assessed by a questionnaire and categorized into three groups: education less than 12 years, 12 years, and academic education.

Weight and height were measured, and body mass index (BMI) was calculated. Age-specific z-scores of height and BMI for girls were computed using 2007 WHO reference data [25].

### Statistical analysis

The normality assumption of each variable was checked using the Kolmogorov-Smirnov test and histogram chart. Baseline characteristics and dietary intakes of girls based on attaining the menarche were determined and compared using Student T-test (for normally distributed variables), Mann-Whitney (for skewed variables), and Chi-square (for categorical variables). Cox proportional hazard regression was used to estimating hazard ratios (HRs) and 95% confidence interval (95%CI) for the occurrence of menarche per one standard deviation (SD) of dietary intakes. Censored date (in days) was calculated from the date of entrance to the study to the date of menarche occurrence or the date of the last follow-up for each participant, whichever came first. The proportionality hazards (PH) assumption was evaluated by correlation tests of Schoenfeld residuals and event time using STATA (version 12; STATA Inc., TX, USA). All proportionality assumptions were generally satisfied. Logistic regression was also used to estimate odds of reaching menarche ≤12 years using the data of girls aged ≥12 years at follow-up (*n* = 161). For both Cox and logistic regression analyses, three models were constructed as: a) unadjusted, b) adjusted for baseline age and BMI Z-score, energy intake, and maternal early menarche (yes/no), and C) additionally adjusted for maternal education (three categories).

Substitution analysis was done to estimate the risk of menarche and early menarche by substituting plant protein for animal protein while holding the total consumption of animal and plant protein constant in the fully adjusted model using the leave-one-out model [26]. Based on the model, one type of protein (animal /plant) was replaced by another while holding total protein intake constant. For example, in substitution of plant protein for animal protein, the multivariable model included plant protein and total protein consumption (animal protein was not included). Dietary intake of animal/plant protein was divided by 10 to generate substitution results for their 10-g dietary intakes.

## Results

The baseline characteristics of the study population are presented in Table 1. The mean ± SD of age at baseline was 9.6 ± 1.9 years (ranges 6–14 years), and 61% of girls reached menarche during the study. The median age at menarche was 12 years, with the range between 9 and 16. Age and height and BMI z-score of girls who attained the menarche were higher than those who did not experience menarche. Dietary intakes were not significantly different between the two groups, except for plant protein and high-fat dairy.

**Table 1:** Baseline characteristics of participants^a^

Variables	Total (***n*** = 241)	Menarche (***n*** = 147)	No menarche (***n*** = 94)	***p***-value^b^
Age	9.64 ± 1.91	10.63 ± 1.49	8.08 ± 1.40	< 0.001
BMI z-score	0.40 ± 1.71	0.70 ± 1.69	−0.07 ± 1.63	0.001
Height z-score	0.51 ± 0.99	0.20 ± 0.99	−0.18 ± 0.94	0.004
Maternal age at menarche	13.6 ± 1.4	13.5 ± 1.5	13.7 ± 1.3	0.312
Maternal early menarche	46 (19.4)	30 (20.7)	16 (17.4)	0.532
Maternal education				
< 12 years	47 (19.7)	32 (21.9)	15 (16.3)	0.215
12 years	137 (57.6)	86 (58.9)	51 (37.2)	
Academic education	54 (22.7)	28 (19.2)	26 (28.3)	
Daily dietary intake				
Energy (kcal)	2328 (1829–3135)	2382 (1900–3269)	2193 (1768–2908)	0.069
Plant protein (% of energy)	5.57 (4.80–6.84)	5.84 (4.92–6.99)	5.35 (4.49–6.42)	0.031
Animal protein (% of energy)	6.95 (5.56–8.98)	6.89 (5.31–8.91)	6.99 (5.76–9.01)	0.459
Total protein (% of energy)	13.10 (11.81–14.28)	13.19 (11.85–14.40)	12.85 (11.74–14.22)	0.469
Fat food (g/1000 kcal)	5.55 (2.68–10.05)	6.29 (2.94–10.32)	5.07 (2.50–9.37)	0.281
Red meat (g/1000 kcal)	4.52 (2.27–8.90)	4.22 (2.31–8.67)	4.96 (2.23–10.23)	0.444
Poultry (g/1000 kcal)	6.58 (3.67–12.18)	6.83 (3.47–12.11)	6.31 (3.82–12.42)	0.797
Fish (g/1000 kcal)	3.06 (1.53–5.65)	3.08 (1.50–5.57)	2.94 (1.66–6.02)	0.928
Organ meat (g/1000 kcal)	0.28 (0.04–0.69)	0.27 (0.05–0.69)	0.29 (0.03–0.69)	0.629
Egg (g/1000 kcal)	5.12 (2.73–8.85)	5.91 (3.03–9.33)	4.81 (2.47–8.33)	0.294
Legumes (g/1000 kcal)	9.65 (4.38–17.25)	9.99 (4.29–17.38)	9.13 (4.56–17.23)	0.829
Low-fat dairy (g/1000 kcal)	94.2 (51.8–149.4)	91.1 (46.1–175.9)	103.5 (56.4–147.0)	0.829
High-fat dairy (g/1000 kcal)	92.4 (38.7–143.8)	83.7 (34.7–130.7)	111.0 (41.7–167.5)	0.035
Total dairy (g/1000 kcal)	203 (138–296)	193 (138–291)	209 (147–299)	0.202

^a^Data was reported as mean ± SD, median (quartile 25-quartile 75), or number (%). ^b^Based on Student T-test and Mann-Whitney test for continuous variables and chi-square for categorical variables

After adjusting for baseline age, BMI, energy intakes, and mother’s early menarche, the adjusted HRs (95% CI) for menarche were 1.40 (95% CI: 1.04–1.88; *p* = 0.028) for poultry, 1.24 (95% CI: 1.03–1.50; *p* = 0.025) for low-fat dairy, 0.65 (95% CI: 0.46–0.93; *p* = 0.018) for plant protein, and 1.44 (95%CI = 1.07–1.94; *p* = 0.015) for animal protein per 1-SD dietary intakes. The association between low-fat dairy and menarche became non-significant after further inclusion of the mother’s educational levels into the model, but the other associations remained significant (Table 2). When plant protein replaced animal protein, the risk of menarche reduced 16% (95%CI: 5–25%; *p* = 0.006) per 10-g dietary intakes after adjusting for all covariates.

**Table 2:** Hazard ratios (95% confidence intervals) for the occurrence of menarche based on 1-standard deviation for dietary intakes of protein-containing food groups

Dietary intakes (g)	Model 1	Model 2	Model 3
Total protein	1.15 (0.99–1.34)	1.28 (0.81–2.03)	1.22 (0.78–1.93)
*p-value*	0.068	0.293	0.385
Animal protein	1.14 (0.94–1.39)	1.44 (1.07–1.94)	1.36 (1.01–1.84)
*p-value*	0.173	0.015	0.042
Plant protein	1.17 (0.99–1.38)	0.65 (0.46–0.93)	0.68 (0.48–0.98)
*p-value*	0.066	0.018	0.040
Fat food	1.10 (0.91–1.32)	1.01 (0.80–1.26)	0.96 (0.77–1.20)
*p-value*	0.314	0.955	0.719
Red meat	1.04 (0.83–1.33)	1.16 (0.90–1.49)	1.12 (0.87–1.44)
*p-value*	0.715	0.248	0.375
Poultry	1.06 (0.79–1.41)	1.40 (1.04–1.88)	1.35 (1.00–1.82)
*p-value*	0.709	0.028	0.049
Fish	1.17 (0.93–1.47)	0.96 (0.75–1.23)	0.93 (0.72–1.20)
*p-value*	0.188	0.750	0.577
Organ meat	1.02 (0.74–1.40)	0.89 (0.63–1.26)	0.86 (0.61–1.22)
*p-value*	0.913	0.518	0.404
Egg	1.09 (0.91–1.31)	1.19 (0.99–1.43)	1.16 (0.97–1.40)
*p-value*	0.334	0.070	0.109
Legumes	1.08 (0.91–1.27)	0.90 (0.74–1.10)	0.95 (0.77–1.18)
*p-value*	0.400	0.316	0.651
Low-fat dairy	1.12 (0.94–1.34)	1.24 (1.03–1.50)	1.20 (0.99–1.46)
*p-value*	0.215	0.025	0.064
High-fat dairy	0.96 (0.80–1.15)	0.88 (0.72–1.07)	0.90 (0.73–1.10)
*p-value*	0.629	0.195	0.307
Total dairy	1.04 (0.87–1.25)	1.07 (0.86–1.33)	1.06 (0.85–1.33)
*p-value*	0.628	0.547	0.576

Model 1: Unadjusted

Model 2: Adjusted for baseline age, BMI Z-score, energy intake, and mother’s early menarche

Model 3: Adjusted for baseline age, BMI Z-score, energy intake, and mother’s early menarche and education

In the unadjusted model, the odds of early menarche increased by 90% and decreased by 41% per 1-SD of poultry and plant protein intakes, respectively. Adjustment for baseline age, BMI, energy intake, mother’s early menarche, and educational level attenuated odds ratio (OR) for poultry (adjusted OR = 1.42, 95%CI: 0.65–3.11; *p* = 0.378), but the association remained significant for plant protein (adjusted OR = 0.39, 95% CI: 0.16–0.96; *p* = 0.040) (Table 3). The odds of early menarche was not significant by substituting animal protein with plant protein in the fully adjusted model.

**Table 3:** Odds ratios (95% confidence intervals) for early menarche based on 1-standard deviation for dietary intakes of protein-containing food groups

Dietary intakes (g)	Model 1	Model 2	Model 3
Total protein	0.80 (0.58–1.10)	0.63 (0.26–1.51)	0.56 (0.22–1.39)
*p-value*	0.167	0.298	0.208
Animal protein	0.98 (0.70–1.39)	1.25 (0.65–2.42)	1.20 (0.62–2.33)
*p-value*	0.928	0.505	0.583
Plant protein	0.59 (0.40–0.88)	0.40 (0.16–0.98)	0.39 (0.16–0.96)
*p-value*	0.010	0.046	0.040
Fat food	0.79 (0.54–1.14)	0.74 (0.46–1.18)	0.77 (0.48–1.24)
*p-value*	0.204	0.207	0.279
Red meat	1.34 (0.85–2.11)	1.84 (0.93–3.62)	1.90 (0.96–3.76)
*p-value*	0.205	0.078	0.067
Poultry	1.90 (1.01–3.55)	1.46 (0.67–3.20)	1.42 (0.65–3.11)
*p-value*	0.046	0.344	0.378
Fish	0.79 (0.51–1.23)	0.86 (0.45–1.64)	0.83 (0.42–1.62)
*p-value*	0.291	0.640	0.583
Organ meat	0.74 (0.39–1.42)	0.63 (0.27–1.47)	0.66 (0.28–1.57)
*p-value*	0.368	0.284	0.348
Egg	0.89 (0.62–1.28)	0.85 (0.53–1.36)	0.85 (0.53–1.38)
*p-value*	0.531	0.490	0.515
Legumes	1.06 (0.75–1.50)	1.19 (0.74–1.91)	1.18 (0.73–1.91)
*p-value*	0.737	0.465	0.489
Low-fat dairy	0.98 (0.72–1.33)	1.05 (0.69–1.59)	1.02 (0.67–1.55)
*p-value*	0.879	0.820	0.928
High-fat dairy	0.91 (0.66–1.26)	1.14 (0.72–1.80)	1.07 (0.66–1.72)
*p-value*	0.568	0.567	0.793
Total dairy	0.92 (0.67–1.25)	1.14 (0.71–1.81)	1.06 (0.65–1.73)
*p-value*	0.596	0.596	0.828

Model 1: Unadjusted

Model 2: Adjusted for baseline age, BMI Z-score, energy intake, and mother’s early menarche

Model 3: Adjusted for, baseline age, BMI Z-score, energy intake, and mother’s early menarche and education

## Discussion

In this prospective study, animal protein intake increased and plant protein decreased the risk of menarche. Considering protein-containing foods, higher intakes of poultry and low-fat dairy increased odds of menarche. The odds of early menarche decreased with higher intakes of plant protein; neither animal protein nor protein-containing foods were associated with odds of early menarche.

Similar to our findings, previous studies concerning the association between different protein intake sources and menarche consistently reported that girls attain menarche earlier by consuming more animal protein during childhood; however, the timing in which animal protein intake may influence menarche is controversial [14–16]. Animal protein during the entire childhood period from ages of 3–5 year, 6–8 years, and 2 years before peak growth velocity in the US girls was a predictor of AAM [14], while animal protein at age 3 and 7 but not 10 years in girls living in South-West England were associated with earlier menarche [16]. In contrast, in the German girls, animal protein intakes at age 5–6 years but not at ages 3–4 years were associated with early menarche [15]. Plant protein intake at the age of 3–5 years was suggested as a predictor of menarche in the US girls, but no significant association was indicated between plant protein intake after the age of 5 years [14] while in the German girls, plant protein intakes at the age of 3–6 years were associated with later age at menarche [15]. Our findings showed that dietary intakes of plant protein at ages between 6 to 14 years were related to menarche.

Interestingly, the percent of animal and plant protein from total energy intakes did not differ across the age groups from early to late childhood, according to Berkey et al. and Gunther et al. studies [14, 15]. In our study, animal protein was about 7% of energy intake, which was lower than that in the US girls (about 9% energy intake) and in the German girls (about 8% of the energy intake) [14, 15]. Besides, the plant protein was 5.6% of energy intakes, which was higher compared to the US girls (3.78% of energy) and the German girls (4.3% of energy) [14, 15].

Regarding evidence on dairy intake and AAM, two studies conducted in the US girls [17, 19]; one of which suggested a higher risk of early menarche in girls with higher intakes of milk at the age of 9–12 years [17], while the other one showed the later attaining menarche in girls with higher frequency intakes of total milk and low-fat milk at ages between 9 to 14 years [19]. In Chilean girls, also higher intakes of low-fat dairy, low-fat milk, and yogurt were associated with later AAM [21]. We have previously reported a higher odds of early menarche in girls who consumed more milk at the age of 4–12 years [18]. However, the other studies could not find any significant association between dairy and menarche [16, 20] .

In our study, the intake of fast food and red meat was not associated with menarche. Contrary to our findings, Jansen et al. reported that red meat intake frequency was inversely associated with AAM [20]. Similarly, in the prospective investigation in the South-West England girls, meat intakes at both 7 years and 3 years were strongly positively related to menarche [16]. Consistent with our results, Carwile et al. could not find any significant association between girls’ red-meat intake at the age of 9–14 years and AAM [19].

Other protein-containing foods such as poultry, fish, egg, and legumes have been less studied in relation to the AAM. In our study, poultry intake was inversely associated with menarche, although Jansen et al. could not show any relation between this food group and menarche among Colombian girls [20]. Fish intake has controversial findings associated with the risk of menarche [16, 20]; we could not find any association between fish intake and menarche. Limited studies have been conducted to investigate eggs and legumes intakes in relation to AAM, which provided non-significant results [16, 20].

The previous studies were more interested in investigating the associations of dietary intakes of protein during early to mid-childhood rather than late-childhood, which is close to the age of menarche, perhaps due to the possible effects of puberty on dietary intakes during the late-childhood periods [27]. Girls enter puberty between the ages of 8 and 13 [28]. The age of girls in our study ranged between 6 and 14 years; one-third of them were ≤ 8 years (mid-childhood), reflecting that most of them had entered puberty. Regardless of the possible effect of puberty on dietary intakes, habitual intakes of children may not be stable from early to late childhood. Therefore it makes it difficult to rely on a one-time point of dietary assessment for investigation on menarche. We also found considerable variability among studies in terms of dietary assessment tools, definitions of each food group, it’s unit (gram, serving, gram/1000 kcal, etc.), and frequency (continuous, one serving/day, one serving/week, etc.). Besides, the substantial differences in the food products and the preferred food items consumption across the populations and food preparation methods render the evidence challenging to interpret collectively.

### Strengths and limitations of this study

Considering different dietary protein sources as exposures, accounting for important covariates including maternal menarcheal age and education, and conducting substitution analysis besides the conventional analyses are the main strengths of the study. Our study does have its limitations; first, a small sample size reduces our ability to conduct subgroup analysis based on participants’ baseline age. Second, because most of our participants entered puberty, the potential effects of each stage of puberty on dietary intakes could not be ruled out.

## Conclusions

In conclusion, as the only one conducted in the Middle East and North Africa region, our study showed that animal protein increased the risk of menarche and plant protein decreased. Of protein-containing food groups, intakes of poultry and low-fat dairy were marginally associated with a higher risk of menarche. The odds of early menarche was reduced with higher intake of plant protein, although none of the protein-containing food groups was related to early menarche. Our findings suggested that partly substitution of animal protein with plant sources during childhood may postpone menarche’s timing.

## Supplementary Information

**Additional file 1.**

## Data Availability

The datasets analyzed during the current study are available from the corresponding author on reasonable request.

## Abbreviations

AAM: Age at menarche
BMI: Body mass index
CIs: Confidence intervals
FFQ: Food frequency questionnaire
HRs: Hazard ratios
OR: Odds ratio
SD: Standard deviation
TLGS: Tehran Lipid and Glucose Study
USDA: United States Department of Agriculture
WHO: World health organization
