# Supplementary material for: Longitudinal association of dietary sources of animal and plant protein throughout childhood with menarche
Source: BMC Pediatr. 2021 Apr 28;21:206. doi: 10.1186/s12887-021-02670-8 (PMC8080383; doi:10.1186/s12887-021-02670-8)
Supplement: Supplementary file 1 — Additional file 1. [file 12887_2021_2670_MOESM1_ESM.docx]

**Longitudinal association of dietary sources of animal and plant protein throughout childhood with menarche**

**Nazanin Moslehi^1^, Golaleh Asghari^1^, Parvin Mirmiran^1,2^, Fereidoun Azizi ^3^**

^1^Nutrition and Endocrine Research Center, Research Institute for Endocrine Sciences, Shahid Beheshti University of Medical Sciences, Tehran, Iran

^2^ Department of Clinical Nutrition and Dietetics, Faculty of Nutrition and Food Technology, National Nutrition and Food Technology Research Institute, Shahid Beheshti University of Medical Sciences, Tehran, Iran

^3^ Endocrine Research Center, Research Institute for Endocrine Sciences, Shahid Beheshti University of Medical Sciences, Tehran, Iran

**Correspondence to:**

**Parvin Mirmiran**

Postal address: No. 7, Arghavan-e-gharbi St., Farahzadi Blvd., Shahrak-e-qods, Tehran, Iran. P.O.Box: 19395-4741. Tel: +982122357484. Email: [mirmiran@endocrine.ac.ir](mailto:mirmiran@endocrine.ac.ir)

**Golaleh Asghari**

Postal address: No. 24, Shahid Arabi St, Yemen Blvd, Chamran Exp, Tehran, Iran. P.O.Box: 19395-4763.Tel: + 98 21224 32500. Email: [g_asghari@hotmail.com](mailto:g_asghari@hotmail.com)

**Supplementary file 1**

In the Name of God

Research Institute for Endocrine Sciences

Shahid Beheshti University of Medical Sciences,

Food Frequency Questionnaire (FFQ) "Tehran Lipid and Glucose Study"

Name: Code: Age:

| NO. | Food items | Portion size | How often | | | | Comments |
| --- | --- | --- | --- | --- | --- | --- | --- |
|  |  |  | Daily | Weekly | Monthly | Yearly |  |
| 1 | Lavash bread | 1 slice |  |  |  |  |  |
| 2 | Barbari bread | 1 slice |  |  |  |  |  |
| 3 | Sangak bread | 1 slice |  |  |  |  |  |
| 4 | Taftoon bread | 1 slice |  |  |  |  |  |
| 5 | Baguette bread | 1 small |  |  |  |  |  |
| 6 | Toasted bread | 1 slice |  |  |  |  |  |
| 7 | Cooked rice | 1 plate |  |  |  |  |  |
| 8 | Cooked pasta | 1 plate |  |  |  |  |  |
| 9 | Potato | 1 medium |  |  |  |  |  |
| 10 | French fries | 10 number |  |  |  |  |  |
| 11 | Baked vermicelli (soup noodle) | 1 cup |  |  |  |  |  |
| 12 | Ash noodle (Reshteh) | 1 cup |  |  |  |  |  |
| 13 | wheat flour | 1 cup |  |  |  |  |  |
| 14 | Cookies | 1 number |  |  |  |  |  |
| 15 | Crackers | 1 number |  |  |  |  |  |
| 16 | Yazdi cake | 1 number |  |  |  |  |  |
| 17 | Homemade cake | 1 medium slice |  |  |  |  |  |
| 18 | Other cakes | 1 number |  |  |  |  |  |
| 19 | Corn | 1 medium |  |  |  |  |  |
| 20 | Barley | 1 tsp |  |  |  |  |  |
| 21 | Bulgur | 1 cup |  |  |  |  |  |
| 22 | Lentil | 1 cup |  |  |  |  |  |
| 23 | Beans | 1 cup |  |  |  |  |  |
| 24 | Chickpea | 1 cup |  |  |  |  |  |
| 25 | Broad bean | 1 cup |  |  |  |  |  |
| 26 | Soy bean | 1 cup |  |  |  |  |  |
| 27 | Mung bean | 1 cup |  |  |  |  |  |
| 28 | Split chickpea | 1 cup |  |  |  |  |  |
| 29 | Beef | 1 slice |  |  |  |  |  |
| 30 | Lamb meat | 1 slice |  |  |  |  |  |
| 31 | Ground beef | 1 tablespoon |  |  |  |  |  |
| 32 | Chicken with skin | 1 medium piece |  |  |  |  |  |
| 33 | Chicken without skin | 1 medium piece |  |  |  |  |  |
| 34 | Fish (except tuna) | 1 medium piece (one palm full) |  |  |  |  |  |
| 35 | Tuna (canned) | 1/2 cans |  |  |  |  |  |
| 36 | Hamburger | 1 number |  |  |  |  |  |
| 37 | Sausage | 1 number (Germany * cocktails *) |  |  |  |  |  |
| 38 | Beef ham | 1 slice |  |  |  |  |  |
| 39 | Heart, liver and kidney | 1 slice |  |  |  |  |  |
| 40 | Egg | 1 number |  |  |  |  |  |
| 41 | Tripe and Rennet | 1 piece |  |  |  |  |  |
| 42 | Tongue | 1 whole number |  |  |  |  |  |
| 43 | Brain | 1 whole number |  |  |  |  |  |
| 44 | Kalle-Kind of organ meat | 1 number |  |  |  |  |  |
| 45 | Pache-kind of organ meat | 1 number |  |  |  |  |  |
| 46 | Pizza | 1 slice |  |  |  |  |  |
| 47 | Skimmed milk | 1 cup |  |  |  |  |  |
| 48 | Low-fat milk (< 2%) | 1 cup |  |  |  |  |  |
| 49 | Whole milk (> 2%) | 1 cup |  |  |  |  |  |
| 50 | Cacao milk | 1 cup |  |  |  |  |  |
| 51 | Chocolate milk | 1 cup |  |  |  |  |  |
| 52 | Concentrated yogurt | 1 tablespoon |  |  |  |  |  |
| 53 | Plain yogurt | 1 cup |  |  |  |  |  |
| 54 | Full fat yogurt | 1 cup |  |  |  |  |  |
| 55 | Cream yogurt | 1 tablespoon |  |  |  |  |  |
| 56 | Cheese | 1 slice |  |  |  |  |  |
| 57 | Cream cheese | 1 slice |  |  |  |  |  |
| 58 | Dough | 1 cup |  |  |  |  |  |
| 59 | Cream | 1 tablespoon |  |  |  |  |  |
| 60 | Traditional ice cream | ½ cup |  |  |  |  |  |
| 61 | Non-traditional ice cream | ½ cup |  |  |  |  |  |
| 62 | Butter | 1 slice |  |  |  |  |  |
| 63 | Margarine | 1 slice |  |  |  |  |  |
| 64 | Kashk | 1 tablespoon |  |  |  |  |  |
| 65 | Shredded lettuce | 1 cup |  |  |  |  |  |
| 66 | Tomato | 1 medium |  |  |  |  |  |
| 67 | Cucumber | 1 medium |  |  |  |  |  |
| 68 | Fresh Herbs | 1 small plate |  |  |  |  |  |
| 69 | Cooked greens | 1 cup |  |  |  |  |  |
| 70 | Pumpkin | 1 medium |  |  |  |  |  |
| 71 | Squash | 1 medium |  |  |  |  |  |
| 72 | Eggplant | 1 medium |  |  |  |  |  |
| 73 | Celery | 1 cup |  |  |  |  |  |
| 74 | Green peas | 1 cup |  |  |  |  |  |
| 75 | Green beans | 1 cup |  |  |  |  |  |
| 76 | Raw carrots | 1 medium |  |  |  |  |  |
| 77 | Cooked carrots | 1 medium |  |  |  |  |  |
| 78 | Garlic | 1 clove |  |  |  |  |  |
| 79 | Raw onion | 1 small |  |  |  |  |  |
| 80 | Fried onions | 1 tablespoon |  |  |  |  |  |
| 81 | Cabbage varieties | 1 cup |  |  |  |  |  |
| 82 | Bell peppers | 1 medium |  |  |  |  |  |
| 83 | Raw spinach | 20 medium leaves |  |  |  |  |  |
| 84 | Cooked spinach | 1 cup |  |  |  |  |  |
| 85 | Turnip | 1 medium |  |  |  |  |  |
| 86 | Small green pepper | 1 medium |  |  |  |  |  |
| 87 | ketchup | 1 tablespoon |  |  |  |  |  |
| 88 | Pickles in vinegar | 1 tablespoon |  |  |  |  |  |
| 89 | Salted vegetables | 1 tablespoon |  |  |  |  |  |
| 90 | Pickled cucumber | 1 medium |  |  |  |  |  |
| 91 | Cantaloupe | ¼ number |  |  |  |  |  |
| 92 | Melon | 1 medium slice |  |  |  |  |  |
| 93 | Watermelon | 1 medium slice |  |  |  |  |  |
| 94 | Pear | 1 medium |  |  |  |  |  |
| 95 | Apricot | 1 medium |  |  |  |  |  |
| 96 | Cherries | 10 number |  |  |  |  |  |
| 97 | Apple | 1 medium |  |  |  |  |  |
| 98 | Peach | 1 medium |  |  |  |  |  |
| 99 | Nectarine | 1 medium |  |  |  |  |  |
| 100 | Green plum | 1 medium |  |  |  |  |  |
| 101 | Fresh figs | 1 medium |  |  |  |  |  |
| 102 | Dried figs | 1 medium |  |  |  |  |  |
| 103 | Grape | 1 medium bunch |  |  |  |  |  |
| 104 | Kiwi | 1 medium |  |  |  |  |  |
| 105 | Grapefruit | 1 medium |  |  |  |  |  |
| 106 | Orange | 1 medium |  |  |  |  |  |
| 107 | Persimmon | 1 medium |  |  |  |  |  |
| 108 | Tangerine | 1 medium |  |  |  |  |  |
| 109 | Pomegranate | 1 medium |  |  |  |  |  |
| 110 | Date | 1 medium |  |  |  |  |  |
| 111 | Plums (yellow and red) | 1 medium |  |  |  |  |  |
| 112 | Sour cherry | 10 number |  |  |  |  |  |
| 113 | Strawberry | 3 number |  |  |  |  |  |
| 114 | Banana | 1 medium |  |  |  |  |  |
| 115 | Sweet lemon | 1 medium |  |  |  |  |  |
| 116 | Lemon | 1 medium |  |  |  |  |  |
| 117 | Grapefruit juice | 1 cup |  |  |  |  |  |
| 118 | Orange juice | 1 cup |  |  |  |  |  |
| 119 | Apple juice | 1 cup |  |  |  |  |  |
| 120 | Cantaloupe juice | 1 cup |  |  |  |  |  |
| 121 | Cranberry | 1 cup |  |  |  |  |  |
| 122 | Fresh Pineapple | 1 cup |  |  |  |  |  |
| 123 | Canned Pineapple | 1 cup |  |  |  |  |  |
| 124 | Raisins | 1 tablespoon |  |  |  |  |  |
| 125 | Persian cantaloupe | 1 cup |  |  |  |  |  |
| 126 | Fresh mulberry | 10 number |  |  |  |  |  |
| 127 | Dried mulberry | 20 number |  |  |  |  |  |
| 128 | Dried Peach | 10 number |  |  |  |  |  |
| 129 | Dried apricot | 10 number |  |  |  |  |  |
| 130 | Green Olive | 10 number |  |  |  |  |  |
| 131 | Canned fruits | 1 can |  |  |  |  |  |
| 132 | Hydrogenated oils | 1 tablespoon |  |  |  |  |  |
| 133 | Liquid Oil | 1 tablespoon |  |  |  |  |  |
| 134 | Olive oil | 1 tablespoon |  |  |  |  |  |
| 135 | Tallow (fat) | 1 medium slice |  |  |  |  |  |
| 136 | Ghee | 1 tablespoon |  |  |  |  |  |
| 137 | Mayonnaise | 1 tablespoon |  |  |  |  |  |
| 138 | Peanut | 20 number |  |  |  |  |  |
| 139 | Almond | 10 number |  |  |  |  |  |
| 140 | Walnut | 1 number |  |  |  |  |  |
| 141 | Pistachios | 10 number |  |  |  |  |  |
| 142 | Hazelnut | 10 number |  |  |  |  |  |
| 143 | Seeds (watermelon, pumpkin, sunflower) | 1 cup |  |  |  |  |  |
| 144 | Sugar cube | 10 number |  |  |  |  |  |
| 145 | Sugar | 1 teaspoonful |  |  |  |  |  |
| 146 | Honey | 1 teaspoonful |  |  |  |  |  |
| 147 | Jams (by type) | 1 tablespoon |  |  |  |  |  |
| 148 | Soft drinks | 1 cup |  |  |  |  |  |
| 149 | Pastries | 1 medium |  |  |  |  |  |
| 150 | Creamy pastries | 1 medium |  |  |  |  |  |
| 151 | GAZ | 1 medium |  |  |  |  |  |
| 152 | Candy | 1 number |  |  |  |  |  |
| 153 | SOHAN | 1 Piece |  |  |  |  |  |
| 154 | Puff | 1 pack |  |  |  |  |  |
| 155 | Chocolate | 1 number |  |  |  |  |  |
| 156 | Caramel cream | 1 tablespoon |  |  |  |  |  |
| 157 | Tea | 1 cup |  |  |  |  |  |
| 158 | Salt | 1 tablespoon |  |  |  |  |  |
| 159 | Broth(only water) | 1 cup |  |  |  |  |  |
| 160 | Chips | 1 pack |  |  |  |  |  |
| 161 | Coffee | 1 cup |  |  |  |  |  |
| 162 | Lemon juice | 1 teaspoonful |  |  |  |  |  |
| 163 | NABAT | 1 medium |  |  |  |  |  |
| 164 | Mushroom | ½ cup |  |  |  |  |  |
| 165 | Homemade HALVA | 1 tablespoon |  |  |  |  |  |
| 166 | Halva Ardeh | ¼ number |  |  |  |  |  |
| 167 | NOGHL | 10 number |  |  |  |  |  |
| 168 | Donuts | 1 number |  |  |  |  |  |
